# Acquisition of novel ball-related skills associated with sports experience

**DOI:** 10.1038/s41598-021-91120-7

**Published:** 2021-06-28

**Authors:** Hirofumi Sekiguchi, Kentaro Yamanaka, Shigeki Takeuchi, Genki Futatsubashi, Hiroshi Kadota, Makoto Miyazaki, Kimitaka Nakazawa

**Affiliations:** 1 Sports and Health Management Program, Faculty of Business and Information Sciences, Jobu University, 634-1 Toyazukamachi, Isesaki-shi, Gunma, 372-8588 Japan; 2 Graduate School of Life Sciences, Showa Women’s University, 1-7-57 Taishido, Setagaya-ku, Tokyo, 154-8533 Japan; 3 Faculty of Management, Josai University, 1-1 Keyakidai, Sakado-shi, Saitama, 350-0295 Japan; 4 School of Information, Kochi University of Technology, 185 Miyanokuchi, Tosayamada, Kami-shi, Kochi 782-8502 Japan; 5 Department of Computer Science, Faculty of Informatics, Shizuoka University, 3-5-1 Johoku, Naka-ku, Hamamatsu-shi, Shizuoka, 432-8011 Japan; 6 Department of Life Sciences, Graduate School of Arts and Sciences, The University of Tokyo, 3-8-1 Komaba, Meguro-ku, Tokyo, 153-8902 Japan

**Keywords:** Neuroscience, Physiology

## Abstract

Some individuals can quickly acquire novel motor skills, while others take longer. This study aimed to investigate the relationships between neurophysiological state, sports experience, and novel ball-related skill acquisition. We enrolled 28 healthy collegiate participants. The participants’ neurophysiological data (input–output curve of the corticospinal tract) were recorded through transcranial magnetic stimulation. Subsequently, the participants performed a novel motor task (unilateral two-ball juggling) on a different day, after which they reported their previous sports experience (types and years). We found that individuals with more years of experience in ball sports showed faster acquisition of novel ball-related skills. Further, this result was not limited to any single ball sport. Therefore, the acquisition of novel ball-related skills is associated with familiarity with a ball’s nature. Furthermore, gain of the corticospinal tract was negatively and positively correlated with the years of experience in primary ball and non-ball sports (implemented for the longest time in individuals), respectively. These results could be associated with the extent of proficiency in their primary sport. The chosen type of sports (e.g., ball or non-ball) could critically influence the future acquisition of novel motor skills. This study provides important insights regarding how to approach sports and physical activities.

## Introduction

In daily life, especially in physical education classes and/or club activities in school life, there are many opportunities to encounter and practice new motor skills. In such cases, some people can develop new skills quickly, while others take longer. In research terms, we call the former a fast learner and the latter a slow learner. Fast learners are generally considered as “gifted” or “talented,” and their characteristics receive public interest. This difference in motor skill acquisition between fast and slow learners could be attributed to individual differences, including differences in the previous sports experience. Sports experience includes actual exercise (physical activities), action observation, and verbal instructions (including how-to books). Diverse sports experiences have been suggested to increase the motor repertoire^1,2^ and build a foundation for acquiring novel motor skills^3,4^. As evidenced by studies of the motor repertoire with transcranial magnetic stimulation (TMS) and neuroimaging, the corticospinal tract and some brain regions are highly responsive to observations of familiar actions^5–10^. Observing unpracticed or unfamiliar movements, including an artificial hand in action, induces less brain activity than observing real hand actions^11,12^. Therefore, the more diverse the motor repertoire, the better the nervous system responds to the action being observed. This is considered beneficial for motor control and acquiring new motor skills and underscores the importance of various sports experiences. On the other hand, early specialization would provide a greater chance to become exceptional experts in their chosen domain (i.e., sports, music, etc.)^13^. This indicates that continuing a specific sport increases proficiency. Previous studies have reported an association of performance gains with training time (trials or sessions)^14,15^, as well as structural and functional brain alterations with years of experience and weekly training time in specific sports^16^. However, early specialization may rob athletes of the opportunity to try diverse sports. Therefore, we might assume that various sports experiences (i.e., the type or years of practice) are more beneficial for the acquisition of novel motor skills. Several studies have reported neuroplasticity corresponding to a specific sports experience^16,17^, as well as an association of age, sex, and regional brain volume with perceptual motor skill acquisition^18^. However, to the best of our knowledge, no study has investigated the relationship between a wide variety of sports experiences and the acquisition of novel motor skills or neuroplasticity. This study aimed to evaluate the relationship between sports experience and the acquisition of a novel ball-related motor skill (unilateral two-ball juggling). Further, we aimed to examine the relationship between sports experience and corticospinal excitability.

## Results

Twenty-eight healthy collegiate participants with no juggling experience were enrolled in this study. Two participants with extremely high performance levels were excluded because they withdrew in the middle of the motor task due to extreme fatigue. The input–output properties of the corticospinal tract were recorded by TMS to obtain the neurophysiological state of the participants before training; the participants then trained for 250 trials of two-ball juggling with the right hand on a separate day. Finally, after the motor task, the participants documented their sports experiences.

### Relationship between a sports experience and performance

Given that the motor task used in this study involved a ball (i.e., a bean bag ball), in addition to examining the overall sport, we considered non-ball and ball sports experiences separately. Tables 1 and 2 present the non-ball and ball sports experiences, respectively. The participants are arranged in a descending order based on the total number of catches during two-ball juggling. Many participants practiced both types of sports (i.e., non-ball and ball sports) or multiple non-ball and ball sports. Two (Participants A and G) and five (Participants N, S, V, W, and Z) participants lacked non-ball and ball sports experiences, respectively. Eighteen participants practiced more than two sports (including non-ball and ball sports) within the same year (by the age of 12 years). After age 13 years, only five participants practiced more than two sports within a single year. This shows that specialization progressed after the age of 13 years, which is the age that children in Japan join junior high school. Two participants (N and V) began regular sports activities at age 13 years, while two participants (M and Y) lacked regular sports activities after age 16 years. Additionally, five participants (E, H, J, N, and T) lacked regular sports activities after age 19 years. This indicates that the included participants had various sports experiences.

**Table 1:** Experiences (sports event and years) in non-ball sports.

Participants	Athletics	Swimming	Dance	Alpine skiing	Gymnastics	Kendo	Karate	Judo	Wrestling	Badminton
A										
B	9	6								9
C		4								
D	5									
E		6								
F	9	3		2						
G										
H		3					1			
I	6									
J		5								
K	11	2								
L	8									
M		10								
N	6									
O	8									1
P	3									
Q	5							4		
R		7			2					
S	13									3
T									3	
U	6	1								
V	10									
W	8					3				
X	1	2	2			3				
Y		9								
Z	8	5					5			

Each number shows the total years of experience in each sport. Athletics include short-distance running, long-distance running, throwing, and jumping. Kendo is traditional Japanese fencing.

**Table 2:** Experiences (sports event and years) in ball sports.

Participants	Tennis	Soft Tennis	Soccer	Futsal	Baseball	Softball	Basketball	Volleyball	Dodgeball
A	5		18	5	3				
B					12			9	
C							12		
D					3	5			
E								11	
F					5				
G			2				11		1
H			11						
I					6				
J	3						5		
K							3		
L			8	2					
M	5								
N									
O							3		
P	11				7				
Q			7						
R	3	10							
S									
T				10					
U				8					
V									
W									
X	6		3	3	3			3	
Y	2						3		
Z									

Each number shows the total years of experience in each sport.

Initially, the subjects were asked to perform two-ball juggling by their own method without being told the correct method to do it (initial session). During the 10 trials before the practice sessions in the motor task (i.e., initial session), the mean number of catches was 11.8 ± 4.0 (mean ± standard deviation [SD]). This confirmed that all participants were beginners in two-ball juggling. The total number of catches for each individual ranged from 227 to 1683 catches (mean ± SD: 844.8 ± 377.3). For example, catching the ball once per trial over 25 practice sessions would yield 250 catches. One participant failed to catch the ball even once in each trial. Of the 26 participants, the left-handed participant (T) came in 20th place (485 catches), while the two mixed-handed participants ranked 15th (O, 793 catches) and 21st (U, 459 catches). The difference in the total number of catches is indicative of the difference between fast and slow learners.

Table 3 presents the relationships between some indices (gain of the corticospinal tract, number of sports, and sports experiences) and the total number of catches in the two-ball juggling sessions. The gain of the corticospinal tract represents the maximum slope of the input–output curve in the corticospinal tract, which is indicative of an increase rate in corticospinal excitability. There was no significant relationship besides that between ball sports experiences and the total number of catches (*p* < 0.01).

**Table 3:** Coefficient of determination, effect size (Cohen’s *f*^*2*^), and probability in the relationship between indices of sports experiences and total number of catches.

Sports experience indices	Total number of catches		
	*R*^*2*^	Cohen’s *f*^*2*^	*p*
Gain of the corticospinal tract	0.037	0.038	0.350
Number of sports	0.000	0.000	0.994
Number of non-ball sports	0.129	0.149	0.071
Number of ball sports	0.078	0.085	0.166
Sports experiences (years)	0.142	0.165	0.058
Non-ball sports experiences (years)	0.056	0.059	0.245
Ball sports experiences (years)	0.306	0.441	0.003**

**p < 0.01.

**Figure 1:**
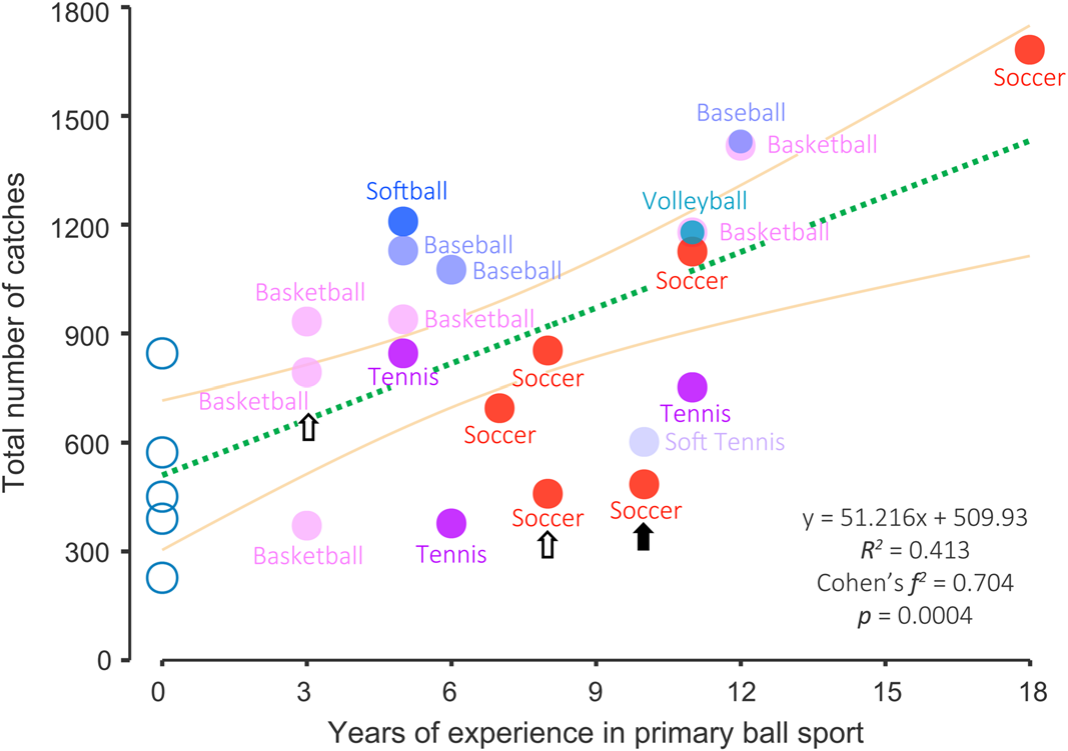
Relationship between years of experience in the primary ball sport and the total number of catches. Black and white arrows indicate the left- and mixed-handed participants, respectively. The overlaid plots can be displayed by resizing.

In Fig. 1, the relationship between primary ball sports experience and the total number of catches is shown as a green dashed line (*F* = 16.9; *df* = 1, 24; *p* = 0.0004). The primary ball sport refers to the ball sport with the longest practice duration by each participant. Approximately 41% of the catches could be explained by the total years of experience in the primary ball sport. That is, variations in the total number of catches could be further explained by limiting to the primary ball sport among ball sports (see Table 3). As indicated by the effect size (Cohen’s *f*^*2*^ = 0.704) in Fig. 1, the relationship between the primary ball sport and the total number of catches was strong. The yellow lines indicate the 95% confidence intervals for the mean estimates. Regardless of the ball size or the limb (upper/lower) handling the ball, the years of experience in the primary ball sport were positively correlated with the total number of catches. There was no significant relationship between years of experience in the primary non-ball sport and the total number of catches (y =  − 40.38x + 1101.1; *R*^*2*^ = 0.12; Cohen’s *f*^*2*^ = 0.14; *F* = 3.27; *df *= 1, 24; *p* = 0.083).

**Figure 2:**
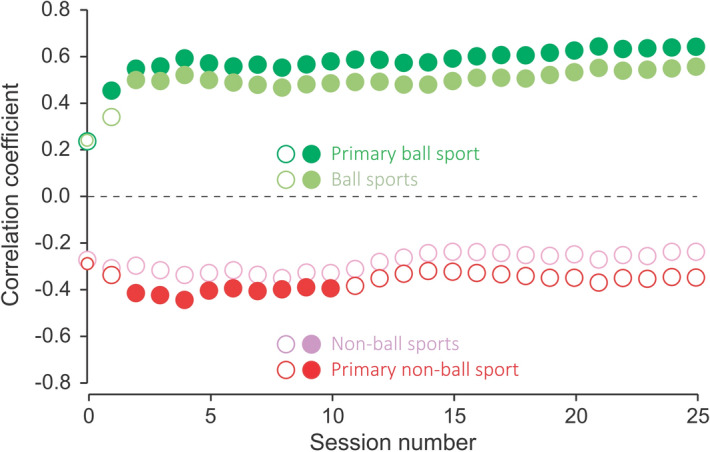
The correlation coefficient for the relationship between years of experience in sports and the total number of catches in each session. Closed and open circles indicate significant and non-significant relationships, respectively. Session zero represents the initial value before the practice session. The overlaid plots can be displayed by resizing.

Figure 2 shows the coefficient of correlation in the relationship between years of experience in sports (ball and non-ball sports; primary ball and primary non-ball sports) and the number of catches in each session. The closed circles indicate a significant relationship (*p* < 0.05). Regarding the positive plots, significant relationships were observed from the 1^st^ or 2^nd^ session (i.e., the first 10 or 20 trials). Regarding the negative plots, there were significant relationships from the 2nd to 10th session, but not in the other sessions, for years of experience in primary non-ball sports.

**Figure 3:**
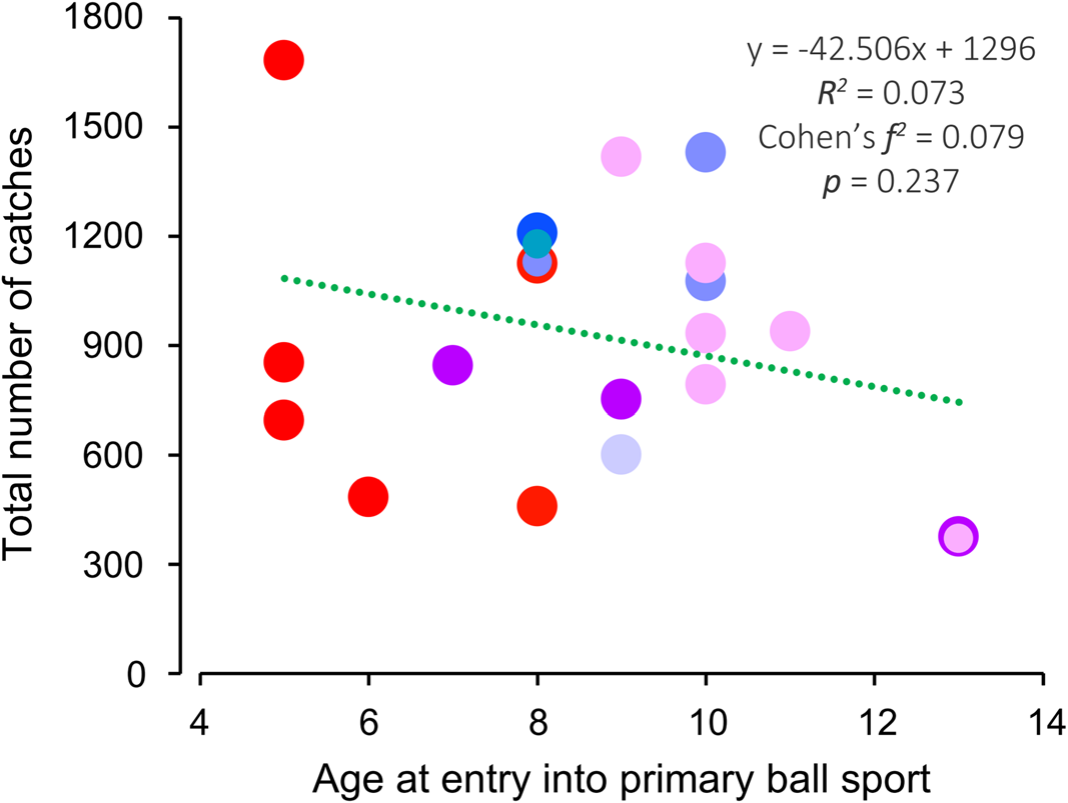
Relationship between the age at entry into the primary ball sport and the total number of catches. The color of each plot corresponds to the identically colored sports in Fig. 1. The overlaid plots can be displayed by resizing.

Figure 3 shows the relationship between the age at entry (5–13 years) into primary ball sports and the total number of catches (*F* = 1.49; *df* = 1, 19; *p* = 0.237). As five participants lacked ball sports experience, we performed analysis using data from 21 participants. Regarding the acquisition of novel ball-related skills, there was no clear tendency in the participants’ age at entry into the primary ball sport. Additionally, individuals who started primary ball sports earlier tended to have longer years of experience (*F* = 4.13; *df* = 1, 19; *p* = 0.056).

### Relationship between sports experience and corticospinal excitability

**Figure 4:**
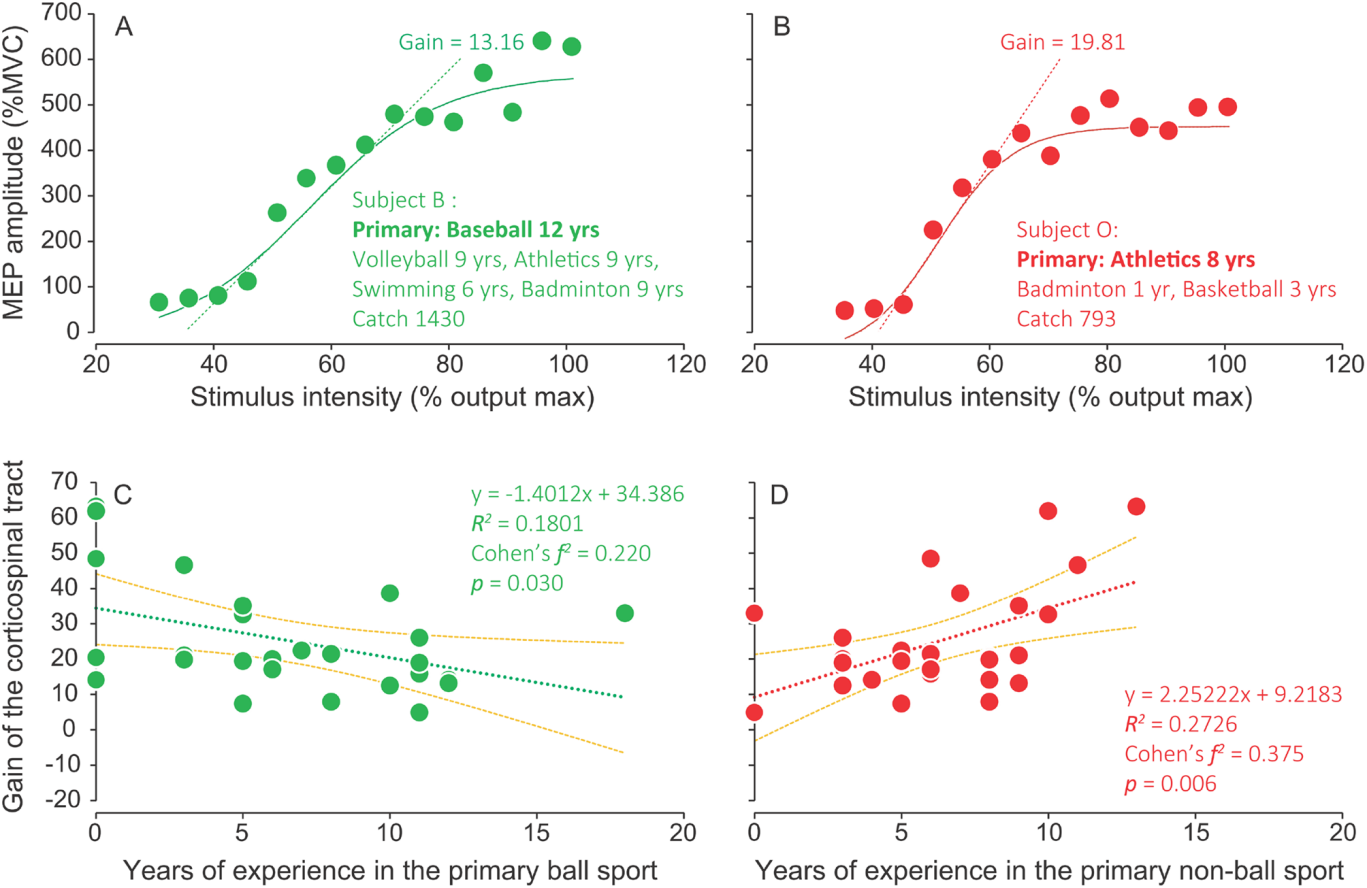
Characteristic relationship between years of sports experience and corticospinal excitability. (**A**,**B**) A typical input–output curve in participants with longer years of experience in primary ball and non-ball sports, respectively. (**C**,**D**) Relationship between years of experience in the primary sport (ball and non-ball) and gain of the corticospinal tract.

In the TMS experiment, background activity (BGA) levels were maintained to approximately 10% of the maximum voluntary contraction (MVC) (11.07 ± 1.80% MVC). Figure 4A,B present a typical input–output curve in participants with longer years of experience in the primary ball (12 years for baseball) and non-ball (8 years for athletics) sports. Figure 4C,D show the relationship between the years of experience in the primary ball or non-ball sport and gain of the corticospinal tract, with the former and latter showing a significant negative (*p* < 0.05) and positive (*p* < 0.01) relationship, respectively. The yellow lines indicate 95% confidence intervals for the mean estimates. Five and two participants had never practiced any ball and non-ball sports, respectively. The remaining 19 (73%) participants had practiced both ball and non-ball sports. There were opposite relationships between years of sports experience and gain of the corticospinal tract for primary ball and non-ball sports. Primary ball sports included basketball (n = 6), soccer (n = 6), baseball (n = 3), tennis (n = 3), soft tennis (n = 1), softball (n = 1), volleyball (n = 1), and no practice (n = 5). Primary non-ball sports included long-distance running (n = 10), swimming (n = 7), throwing (n = 3), jumping (n = 1), short-distance running (n = 1), wrestling (n = 1), kendo (n = 1), and no practice (n = 2).

## Discussion

This study revealed faster acquisition of novel ball-related skills among individuals with more years of experience in their primary ball sports. Although this finding may appear obvious given that a ball was used in the motor task, it was not related to the ball size or the limbs (upper or lower) handing the ball. Contrary to popular belief, there was no association between the number of ball and non-ball sports (i.e., diverse experiences) with the acquisition of novel ball-related skills (see Table 3). Furthermore, skill acquisition was more significantly associated with years of experience in primary ball sports (*p* = 0.0004, Cohen’s *f*^*2*^ = 0.704) than those in all ball sports practiced by each participant (*p* = 0.003, Cohen’s *f*^*2*^ = 0.441). This finding confirms the notion that diverse experiences, even solely in ball sports, are not absolutely essential for the acquisition of novel ball-related skills.

### Years of experience in ball sports and acquisition of novel ball-related skills

In humans, repeated practice enables the improvement of skill and proficiency. Therefore, attaining high proficiency in a single ball sport was considered most beneficial for acquiring novel ball-related skills. As shown in Fig. 2, among-session differences in the number of catches, which depended on the years of experience in ball sports, appeared after the first 10 or 20 trials. This indicates that the years of experience (i.e., the extent of proficiency) in a single ball sport strongly influence the acquisition of novel ball-related skills. However, because the relationship was not restricted to a specific ball sport, a common factor in all ball sports could form the underlying basis for the acquisition of novel ball-related skills. Specifically, gaining familiarity with the nature of a ball could form a base for acquiring novel ball-related skills, with subsequent sophistication across the years of experience. For instance, compared with a less-proficient individual, a proficient individual can better predict the ball trajectory and timing after throwing a ball in the air. In fact, elite basketball players can predict the success of free throws earlier and more accurately than expert watchers (journalists and coaches) or novices^10^. Therefore, proficiency in ball sports, by gaining familiarity with the nature of a ball, can be one of the factors underlying the acquisition of novel ball-related skills.

The observed among-session differences in the relationship between years of experience in primary non-ball sports and the number of catches (Fig. 2) could reflect the effect of years of experience in a primary ball sport. In our study, only five participants lacked ball sports experience. Therefore, even participants with extensive experience in a primary non-ball sport relatively improved their skills based on their years of experience in a primary ball sport. This could have resulted in the aforementioned among-session differences in the relationship.

Furthermore, high proficiency could yield the acquisition of movement automaticity, i.e., the ability to accurately perform a task with minimal attention required^19^. Movement automaticity frees attentional resources for other tasks^20^. A previous study reported similar golf performance between novices without any instruction and those instructed to focus their attention on their arms (i.e., internal focus); however, they were both inferior to participants instructed to focus their attention on the club (i.e., external focus)^21^. Although these are experimental results, they indicate that daily practice can promote motor proficiency and yield movement automaticity, which allows more attention to be directed toward external objects. Real-life examples include expert pianists playing while holding a conversation or elite basketball players dribbling while judging the situation of the enemy and ally. Therefore, individuals sufficiently skilled to achieve movement automaticity in their primary ball sports can utilize their attentional resources to acquire novel ball-related skills, which could allow sufficient attention to external objects (e.g., a ball and/or surrounding environment).

### Effect of age at entry

Ericsson et al. reported that early specialization facilitates exceptional expertise^11^. Compared with late specialization, early specialization leads to more years of experience as long as the individual continues practicing a specific ball sport. In our study, individuals who started early tended to have more years of experience, which could also benefit the acquisition of novel ball-related skills. However, we observed no correlation between the age at entry and acquisition of novel ball-related skills. A previous study reported that elite swimmers with a diverse sports background were part of the national team for longer durations and had longer careers than those with early specialization^22^. Taken together, early specialization is not necessarily a prerequisite for acquiring novel ball-related skills; rather, years of experience, in other words, proficiency, might be crucial for acquiring novel ball-related skills. However, aging is accompanied by deterioration in proprioceptive^23^ and motor function^24–26^. Furthermore, there are individual differences in age-related effects and their onset. Therefore, it should be noted that years of experience do not necessarily yield equal effects across all individuals and generations.

### Association between years of sports experience and gain of the corticospinal tract

Regarding the gain of the corticospinal tract, plastic changes based on practice (years of sports experience) could have occurred or the original gain features could have matched the selected sports, which might be reflected in our findings. Nonetheless, even if our findings are related to the latter case, it has been reported that repeated practice can induce plastic changes^27–30^. Therefore, the present results should partially reflect the neural plasticity in each participant based on their experience. Notably, there were contrasting relationships for years of experience in ball and non-ball sports (see Fig. 4). Specifically, experiences in ball and non-ball sports tended to decrease and increase the gain of the corticospinal tract, respectively. Neuroimaging studies have reported that movement automaticity induces decreased activity in extensive cortical and subcortical regions, including the bilateral cerebellum, rostral supplementary motor area, cingulate motor area, left caudate nucleus, premotor cortex, parietal cortex, and prefrontal cortex^31–34^. Furthermore, movement automaticity alters the connectivity between some brain regions^35,36^. As demonstrated by these neuroimaging studies, movement automaticity is accompanied by alterations in brain activity. Therefore, the observed gain characteristics could be among the functional aspects associated with changes in activities and connectivity in some brain regions over the years.

An animal study reported a positive association of gain in motor neurons with variability in force^37^. If this observation was applied to our findings, the lower gain associated with years of experience in ball sports could contribute to reduced force variability and might facilitate fine motor control. Contrastingly, the higher gain associated with years of experience in non-ball sports could contribute to great power exertion. In particular, sprinting, throwing, martial arts, and other non-ball sports would need greater power rather than fine motor control. A human study^38^ reported that gain of the corticospinal tract in badminton players, which we classified as a non-ball sport, was positively correlated with years of experience. Moreover, the extent of short- and long-interval intracortical inhibition was negatively correlated with the years of experience^38^, which indicates that intracortical inhibition decreases with the years of experience in non-ball sports. Conversely, individuals with more years of experience in ball sports might acquire strong function in intracortical inhibitory circuits involved in reducing unnecessary muscle activation. Another possible explanation for the reduced gain of the corticospinal tract with years of experience in ball sports might be attributed to the effect of phase cancellation. Asynchronous arrival of action potentials at the muscle membrane induces phase cancellation on the surface electromyogram (EMG)^39^. Phase cancellation reduces the motor-evoked potential (MEP) amplitude, which decreases the gain of the corticospinal tract. Kouzaki et al. (2012) suggested that reduced synchronous activity in recruited motor units produced a steadier force output^40^, which could contribute to fine motor control. Contrastingly, increasing gain of the corticospinal tract with years of experience in non-ball sports may result from increased synchronous activity in the recruited motor units. This could yield large MEP amplitudes and cause a higher gain of the corticospinal tract. Therefore, differences in gain between ball and non-ball sports could result from plastic changes dependent on the requirements of each sports characteristic.

We observed no relationship between gain and the total number of catches (see Table 3). Given that the motor task, which involved two-ball juggling with the right arm, requires activation of multiple muscles, the gain in the tested muscle (i.e., the biceps brachii [BB] muscle) represents only one aspect of plastic changes. This could be considered as an experimental limitation. On the other hand, this could be attributed to different motor task strategies given that muscles mainly involved in this motor task might differ across participants. Another possible explanation is that the regions in the central nervous system, other than the primary motor cortex (M1), could be learning sources. For example, during the early stages of motor skill learning, cerebellar-dependent learning mechanisms are required to learn the task dynamics, while the M1 functions are critical during the later stage^41^. Therefore, it might not be possible to determine the direct relationship between gain and the total number of catches in the stage assessed in the present study.

## Conclusion

This study observed a positive correlation between years of experience in a single ball sport with the acquisition of novel ball-related skills. Notably, the fast acquisition of novel ball-related skills was not associated with the ball size or the limb handling the ball, which indicates that this skill acquisition was not specific to any one ball sport. Sports involve skill types other than ball-related skills. Therefore, it might be important to practice various sports to facilitate the acquisition of various novel motor skills. However, our findings suggest that years of experience, that is, the proficiency level, is a crucial factor in the quick acquisition of novel motor skills.

## Methods

### Participants

This study recruited 28 healthy collegiate participants (22 males and 6 females, 19.8 ± 1.0 years) without a history of neurological disorders. We chose this age group since it does not show significant aging effects and has relatively diverse sports experiences. Based on the Edinburgh Handedness Inventory, the participants were classified as right-handed (n = 25), left-handed (n = 1), and mixed-handed (n = 2)^42^. The score on this inventory is expressed as a laterality quotient (mean ± SD, 81.8 ± 42.7), which ranges from + 100 (right-handedness in all tasks) to − 100 (complete left-handedness). This study was approved by the biological ethics committee of Jobu University (approval no: 16-B04) and was conducted in accordance with the Declaration of Helsinki. Moreover, all participants provided informed consent before enrollment. All experiments were performed in accordance with approved guidelines and regulations.

### TMS study

TMS was conducted with the participants seated and facing an oscilloscope positioned approximately 50 cm away. The right elbow joint was flexed to 90 degrees (full extension = 0 degrees); further, the upper arm was placed along the body and the forearm was supinated. An EMG of the right BB muscle was recorded using bipolar Ag/AgCl surface electrodes (10 mm diameter; Vitrode F-150S, Nihon Kohden Corporation) placed over the muscle belly. The ground electrode was attached to the acromial process in the shoulder. The EMG signal was amplified (1000 times) and band-pass filtered (5.3–3 kHz) using a bioamplifier (AM-601G, Nihon Kohden Corporation) before sampling (2 kHz) with an analog-to-digital (A/D) converter (Micro 1401 MkII, CED). The participants exerted the MVC by producing an isometric force for lifting the inferior table surface. During the TMS session, the participants exerted EMG activity levels corresponding to 10% MVC in the same manner. TMS was applied to the scalp using a stimulator (MagStim Co., Whitland, Dyfed, UK) with a maximum magnetic field strength of 2.2 T and a double 70-mm stimulating coil. A figure-of-eight coil was held over the left motor cortex (at the optimum scalp position for eliciting motor responses in the contralateral BB muscle) with the induced current flowing in a posteroanterior direction. The focal point was defined as the lowest threshold site that yielded a response specifically in the BB muscle at a BGA level of approximately 10% MVC. The motor threshold (MT) was defined as the minimum stimulus intensity that produced a liminal motor-evoked response (about 50–100 mV in 50% of trials). Stimulus intensity was expressed as a percentage of the maximum stimulator output and was increased at intervals of 5% of the maximal output from 10% below the MT to the plateau response level. The order of stimulus intensities was previously reported to not affect the response^43,44^. Each stimulus intensity was repeated thrice. Stimuli were randomly delivered at 8–10 s intervals. The direct current component was subtracted from the EMG data; further, the peak-to-peak amplitude of MEPs were evaluated by normalizing with the mean MVC amplitude. We evaluated the maximum slope of the input–output curve of the corticospinal tract. The maximum slope was calculated by fitting the Boltzmann sigmoidal curve to the relationship between stimulus intensity and MEP size. The Boltzmann sigmoidal function was fitted to the data points using the Levenberg–Marquardt non-linear least-mean-square algorithm^45^. The Boltzmann equation relating MEPs and stimulus intensity (S) were calculated as follows:

$${\text{MEP}}(S) = \frac{{{\text{MEP}}_{(\max )} }}{{1 + e\left( {\frac{{S_{50} - S}}{k}} \right)}}$$

This equation has three parameters: the maximum value (MEP_max_), the stimulus intensity (*S*_50_) required to obtain a response of 50% of the maximum, and the slope parameter *k*. The inverse of the slope parameter (1/*k*) is directly proportional to the maximal steepness of the function, which occurs at *S*_50_. We directly calculated the maximum slope by differentiating the input–output sigmoidal curve equation, which was defined as the gain of the corticospinal tract. Regarding the gain of the corticospinal tract, the gain reflects the subliminal fringe size of all neurons in the corticospinal tract, i.e., cortical neurons, spinal interneurons, and motor neurons; further, it indicates the recruitment gain of the corticospinal tract^44,46^. A high and low gain produces a large and small output (i.e., muscle output), respectively, against the input (i.e., motor command).

### Motor task

The novel motor task involved two-ball juggling using the right hand in a rolling-out pattern, with the balls being thrown in an arc to the right (clockwise when viewed from the participants’ perspective). These tasks were performed using bean bag balls, which are commonly used for juggling. A trial was considered complete if the participant stopped moving or dropped a ball. Each session comprised 10 trials; moreover, all but 2 participants performed 25 sessions, with one quitting at the 7th trial in the 21st session and the other at the 1st trial in the 22nd session. Two participants became proficient at juggling and extensively performed the task within single trials that they had to stop halfway through the session due to extreme fatigue.

Before commencing the sessions, the participants were asked to juggle two balls using their right hand across 10 trials without any instruction to confirm that no participants had practiced the motor task before the experiment. Subsequently, the participants watched an exemplar video (approximately 20 s) twice, followed by the first session. To mitigate the effects of fatigue, a 3-min break was taken at 10-session intervals. Additionally, each participant was allowed a 1-min break upon request.

### Sports experiences

The participants were asked to write down all sports (types and years of practice) they had regularly participated in during childhood, elementary school, junior high school, high school, and university (current status). We listed all sports with a participation duration of at least 1 year.

### Statistical data analysis

Both aforementioned participants who quit the juggling practice midway were excluded from the analysis as outliers since the total number of catches exceeded the mean plus two times the standard deviation. Despite quitting midway, their respective total numbers of catches were 2223 and 2753. Simple linear regression analysis was performed to examine the association of the number of sports types or years of experience in sports with the total number of catches while juggling. For further analysis, both factors were subdivided into non-ball and ball sports. Subsequently, we assessed the association between these indicators and the total number of catches while juggling using simple linear regression analysis. Moreover, we investigated the association of the total number of catches or years of experience in primary ball or non-ball sports with gain of the corticospinal tract. The primary sport referred to the sport that the participant had practiced for the longest duration in each type (i.e., ball and non-ball). Statistical analyses were performed using the Jamovi program (Jami project (2020), Version 1.2.27.0, retrieved from https://www.jamovi.org). Statistical significance was set at *p* < 0.05.

## Data Availability

The datasets analyzed during the current study are available from the corresponding author on reasonable request.
